# Cortical kainate receptors and behavioral anxiety

**DOI:** 10.1186/s13041-017-0297-8

**Published:** 2017-05-18

**Authors:** Min Zhuo

**Affiliations:** 1 Center for Neuron and Disease, Frontier Institutes of Science and Technology, Xi’an Jiaotong University, Xi’an, Shanxi 710049 China; 2 Department of Physiology, Faculty of Medicine, University of Toronto, Medical Science Building, Room #3342, 1 King’s College Circle, Toronto, ON M5S 1A8 Canada

## Abstract

The study of glutamatergic synapses mainly focuses on the memory-related hippocampus. Recent studies in the cortical areas such as the anterior cingulate cortex (ACC) show that excitatory synapses can undergo long-term plastic changes in adult animals. Long-term potentiation (LTP) of cortical synapses may play important roles in chronic pain and anxiety. In addition to NMDA and AMPA receptors, kainate (KA) receptors have been found to play roles in synaptic transmission, regulation and presynaptic forms of LTP. In this brief review, I will summarize the new progress made on KA receptors, and propose that ACC synapses may provide a good synaptic model for understanding cortical mechanism for behavioral anxiety, and its related emotional disorders.

## Glutamate and KA receptors

Glutamate mediates the majority of excitatory synaptic transmission in mammalian brains. Both ionotropic and metabotropic glutamate receptors contribute to synaptic transmission, plasticity, as well as modulation. KA receptors are one of three subtypes of ionotropic receptors for glutamate and are composed of five different subunits: GluK1-5 (or called GluR5, GluR6, GluR7, KA1 and KA2). Postsynaptic KA receptors can contribute to fast synaptic responses in different regions of the brain, including spinal cord dorsal horn and cortical areas [1–3], although the majority of postsynaptic currents are carried out by AMPA receptors. In addition to a postsynaptic contribution, KA receptors also regulate the release of excitatory or inhibitory neurotransmitters through presynaptic receptors [4–6]. Furthermore, KA receptors are also reported to be involved in both long-term synaptic plasticity in different regions of brain, including the amygdala and cortex [7]. In this review, I will focus on recent literature on anxiety-related cortical areas, and some comparisons will be made in regard to KA roles in the amygdala.

## Cortical contribution to anxiety

Among several brain regions studied in anxiety, the amygdala is a key structure receiving the most attention. Anxiety is often thought of as an innate fear, the structural basis of anxiety resides in the neural circuitry related to fear response. The amygdala is a key structure for processing neuronal inputs from other parts of the brain, initiating output signals to responding nuclei, and generating various physiological responses, including behavioral, autonomic, and hormonal responses related to anxiety [8–10]. Electrical stimulation of the amygdala in both humans and animals elicits anxiety. Consistently, lesions of the amygdala impair the perception of fear. The efferents from the central amygdala go to the periaqueductal gray (PAG), brainstem, and hypothalamus, which initiate fear-related behavioral, autonomic, and hormonal responses. LTP of synaptic transmission in the basolateral amygdala has been proposed for the key synaptic mechanism for fear memory, while the synaptic basis for anxiety is less understood [11, 12].

In addition to the amygdala, animal and human studies show that different cortical regions have also been indicated in anxiety or anxiety-related behaviors. For example, the ACC and insular cortex (IC), two major brain areas for unpleasantness and emotion, have been demonstrated to play important roles in various types of anxiety [13–17]. The ACC/IC have been implicated in anxiety and fear in both human and animal studies [18–21]. Human imaging studies observed increased ACC and/or IC activity in patients with anxiety disorders [22]. For example, the ACC shows greater activation in patients with social anxiety disorders [23], and activation of the IC has been reported in phobia subjects [24].

## KA receptor mediated synaptic transmission in the cortex

**Fig. 1:**
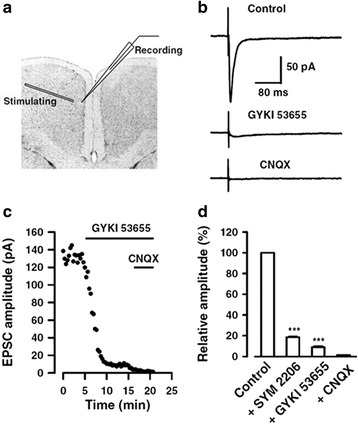
KA receptor-mediated EPSCs in adult ACC pyramidal neurons. **a** Diagram showing the placement of stimulating and recording electrodes in the ACC. **b** Control EPSCs were recorded in the presence of picrotoxin (PTX, 100 μM) and AP-5 (50 μM). After the perfusion of GYKI 53655 (100 μM), a small residual current remained, which could be totally blocked by CNQX (20 μM). In the following example of EPSCs, each trace represents an average of 5–10 consecutive recordings. **c** Sample points showing the time course of GYKI 53655 and CNQX effects on the neuron shown in **b. d** Statistical results showing the percentage of EPSCs in the presence of SYM 2206, GYKI 53655 or CNQX. Modified from Wu et al. [3]

**Fig. 2:**
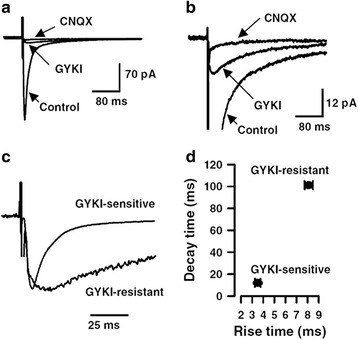
KA receptor-mediated EPSCs show slower kinetics. **a** Superimposed traces showing control EPSCs, EPSCs after application of GYKI 53655, or CNQX. **b** Enlarged traces showing the traces in A. Peak current of control EPSCs is off-scale to emphasize the small GYKI-resistant EPSC. **c** Scaled traces showing the different kinetics for GYKI-sensitive and -resistant current. **d** statistical results for the rise time and decay time constant of GYKI-sensitive and -resistant current. Modified from Wu et al. [3]

In situ hybridization and immunostaining results show that KA receptor subunits are widely expressed in the cortex [25–27]. For example GluK1-3 and GluK5 are highly expressed, whereas GluK4 is either weakly detectable during postnatal days or not expressed. Results from in situ hybridization and immunostaining show that KA receptor subunits are expressed in the ACC [26]. Although KA receptors have been reported in many central synapses, the contribution of KA receptors to baseline synaptic transmission is highly limited. In many brain regions, baseline synaptic transmission is mainly mediated or exclusively mediated by AMPA receptors. Since early reports of KA receptors in nociceptive transmission in the spinal cord dorsal horn [1], KA receptor mediated synaptic responses have also been reported in ACC and IC [3, 28]. Single shock stimulation could induce small KA receptor-mediated excitatory postsynaptic currents (EPSCs) (see Fig. 1). KA receptor EPSCs had a significantly slower rise time course and decay time constant compared with AMPA receptor-mediated EPSCs (Fig. 2). High frequency repetitive stimulation significantly facilitated KA receptor EPSCs. Studies using genetically modified mice with the deletion of GluK1 (GluR5) and/or GluK2 (GluR6) show that both GluK1 and GluK2 are involved in synaptic transmission in the adult ACC. KA EPSCs are ~5–10% of AMPA/KA EPSCs in all layers of the adult mouse and rat insular cortex [2]. Similar to the ACC, genetic deletion of GluK1 or GluK2 subunit partially reduced postsynaptic KA EPSCs, and exposure of GluK2 knockout mice to the selective GluK1 antagonist UBP 302 could significantly reduce the KA EPSCs. These data suggest that both GluK1 and GluK2 play functional roles in the IC.

## KA receptor mediated synaptic transmission in the amygdala

In the amygdala, mRNA for GluK1,2 and GluK5 subunits are highly expressed Among them, GluK1 is highly expressed. It has been reported that KA receptors contribute to postsynaptic excitatory responses of basolateral amygdala induced by the stimulation of external capsule [29]. Interestingly, this excitatory response (about 30% of total current) is sensitive to the inhibition of GluK1 receptor antagonist. Due to the slow decay kinetics of KA mediated currents, the postsynaptic responses summate in response to high frequency stimulation.

## KA receptor and synaptic regulation

In the basolateral amygdala, presynaptic GluK1 is shown to bidirectionally modulate GABA release, possibly due to different types of GluK1-containing KA receptors with different agonist affinities [7]. In vitro slice recording showed that GluK1 is selectively expressed in interneurons, and its activation could largely depolarize those interneurons and increase synaptic GABA release as well as GABA tonic currents. More importantly, the GluK1 activation in the basolateral amygdala reduced the output to the central amygdala, which may explain the increased anxiety phenotype in the GluK1 knockout mice [6] In addition, the regulation of excitatory glutamate transmission by KA receptors have been also reported in the amygdala [30, 31], and such regulation is also mixed (inhibition and facilitation).

In the ACC, activation of GluK1 also triggers action potential-dependent GABA release, which is also required for the activation of voltage-dependent calcium channel and Ca^2+^ influx (Fig. 4). The effect of GluK1 activation is selective to the GABAergic, but not glutamatergic synaptic transmission [32]. Interestingly, we found that GluK2 knockout mice showed reduced fear memory but not anxiety, which may be due to its role in the induction of LTP in the lateral amygdala [33].

## KA receptor and LTP in the cortex and amygdala

There are at least three major forms of LTP in central synapses [34, 35]. The first type is post-LTP. The second one is pre-LTP. The last one is pre-LTP that requires diffusible messengers [34]. In general, NMDA receptor dependent LTP takes place in most of central synapses, in addition to the hippocampus [36]. In the ACC, there are at least two forms of LTP. The post-synaptic form of LTP requires activation of NMDA receptors. This form of LTP is independent of KA receptors. The expression of LTP is likely due to increases in AMPA receptor mediated functions, including phosphorylation of GluA1 AMPA receptors [37]. There is no involvement of KA receptor in post-LTP.

**Fig. 3:**
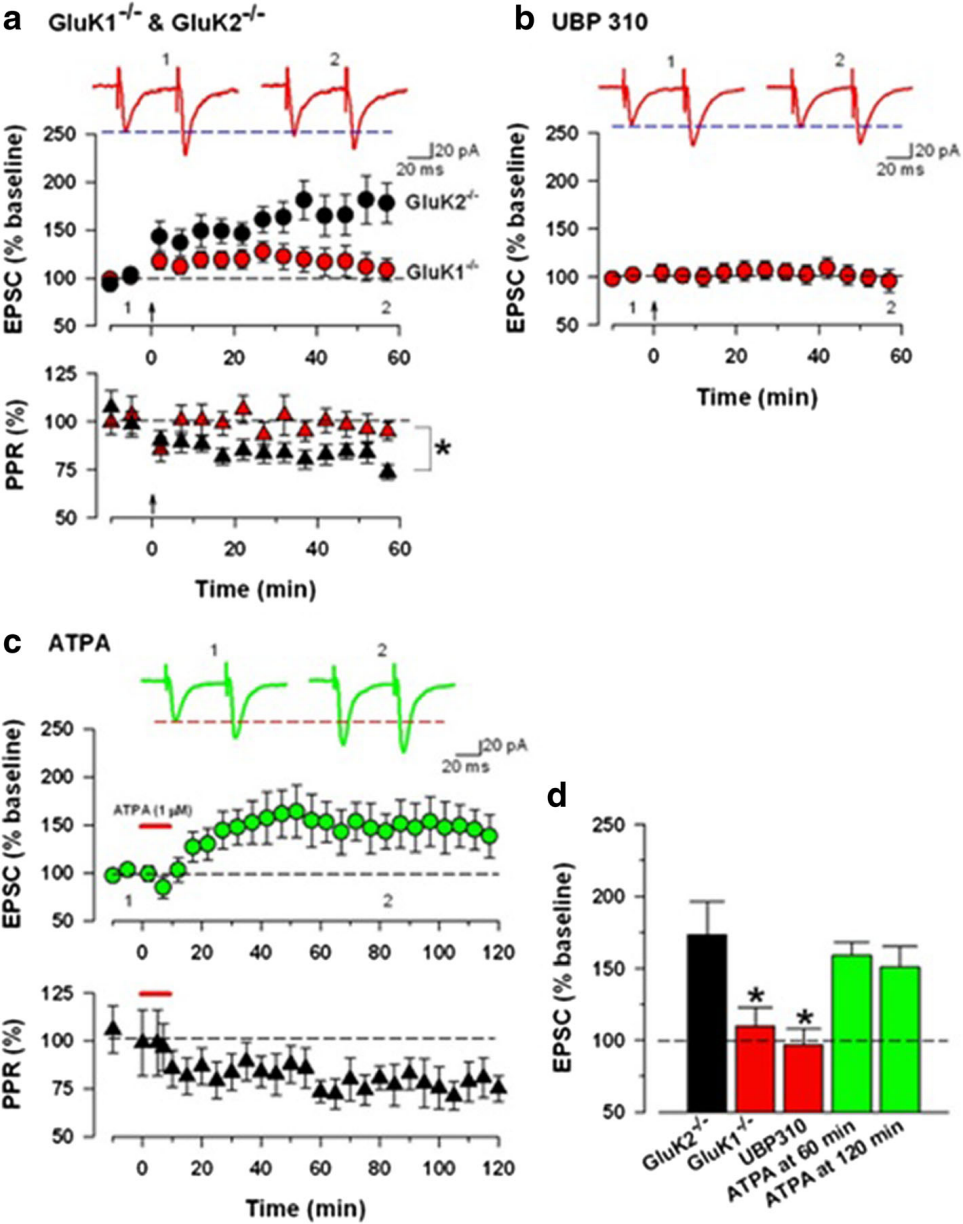
KA receptors mediate the induction of pre-LTP. **a** *Upper panel*: In GluK1-/- mice, sample traces of eEPSCs with paired-pulse stimulation at 50 ms during baseline (1) and 60 min after the induction stimulus (2) at a holding membrane potential of -60 mV. *Middle panel*: GluK2-/-mice showed normal pre-LTP (black circle). GluK1-/- mice showed greatly reduced pre-LTP (*red circle. Bottom panel*: PPR for the GluK2-/- (*black*) and GluK1-/-(*red*) groups. **b** A specific GluK1 antagonist, UBP310 (10 μM), completely blocked pre-LTP. **c** *Upper panel*: A GluK1 agonist, ATPA (1 μM for 10 min), induced long lasting potentiation, recorded for 2 h. *Bottom panel*: Averaged data of PPR change before and after ATPA application. **d** Summary of the effects of GluK2-/-, GluK1-/-, a GluK1 antagonist or a GluK1 agonist on pre-LTP. The amplitudes of eEPSCs in GluK1-/- or UBP310 groups were significantly decreased compared with control pre-LTP. There was no difference among control pre-LTP, GluK2-/- and ATPA. Modified from Koga et al [38]

**Fig. 4:**
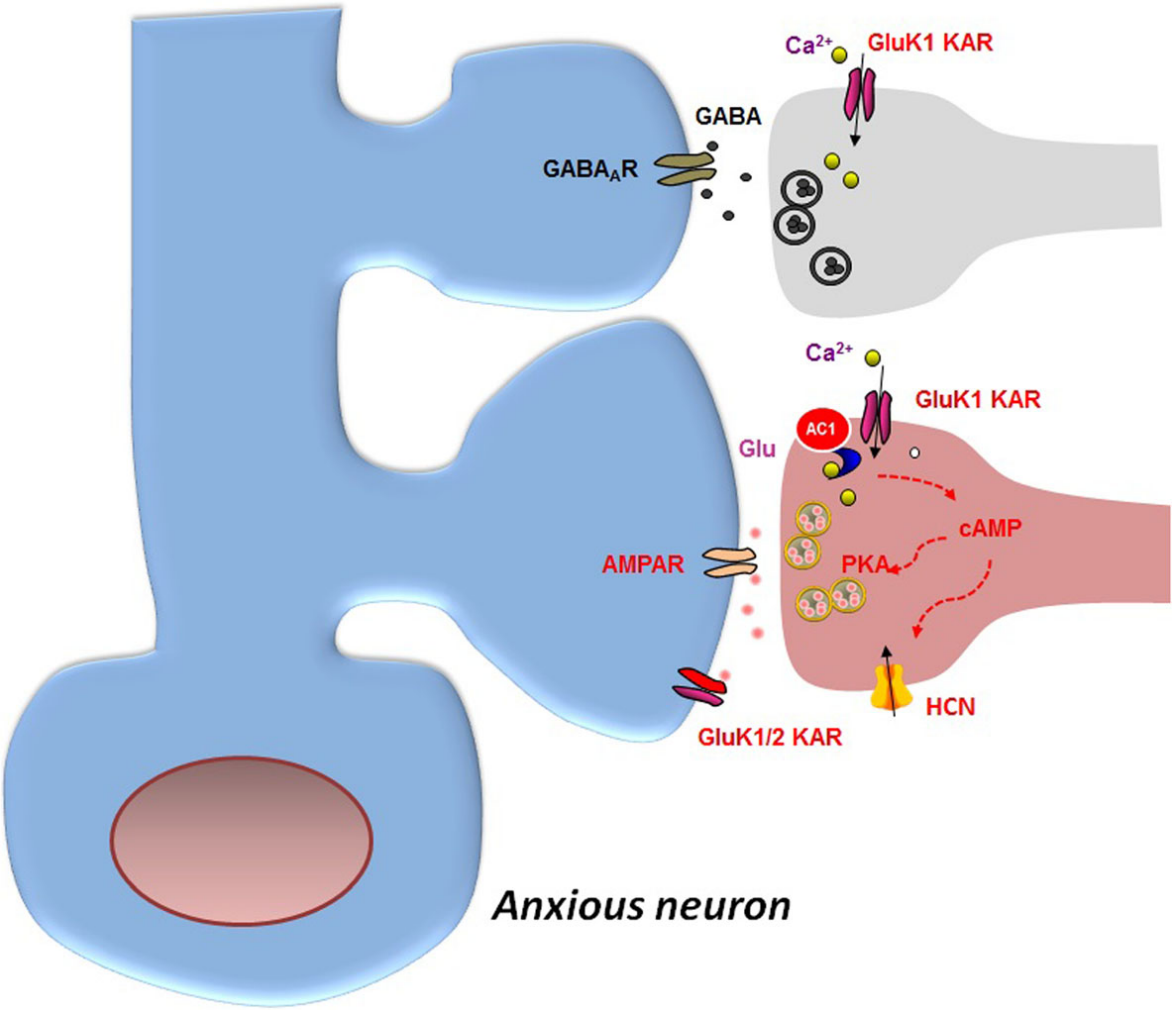
KA receptors contributes to the regulation of synaptic transmission and presynaptic LTP in cingulate cortex. At excitatory synapses in the ACC (*left side*, *lower panel*), glutamate mediates excitatory transmission, and postsynaptic KA receptors (GluK1 and 2) mediate some of postsynaptic response in addition to AMPA receptors. GluK1 containing KA receptors also locate at presynaptic terminals, and its activation contributes to presynaptic form of LTP. The presynaptic Ca^2+^ influx via GluK1 KARs leads to activation of the AC1-PKA pathway, which then results in modulation of HCN channels resulting in a long-lasting increase in glutamate release. At inhibitory synapses in the ACC (*left side*, *top panel*), presynaptic GluK1 KA receptors also regulate the release of GABA in a biphasic manner. The exact signaling mechanism remains to be investigated though

Another form of LTP, pre-LTP, has been recently identified [38–40] (see Figs. 3 and 4). It requires the activation of KA GluA1 receptor. Activation of GluK1-containing KARs appears sufficient for the induction of pre-LTP in the ACC. Together, these results suggest that presynaptic GluK1 receptors are both necessary and sufficient for the induction of pre-LTP in the ACC.

In the amygdala, KA receptor dependent LTP has been reported. Repetitive low frequency stimulation induce LTP in the basolateral amygdala, and this form of LTP is NMDA receptor independent, and sensitive to inhibition of GluK1 receptor [41]. Moreover, this form of LTP is not synapse selective and can be heterosynaptic. NMDA receptor dependent form of LTP, however, can be used using the pairing protocol in the same regions. This form of LTP does not require KA receptors. However, there is evidence that presynaptic KA receptor may contribute to postsynaptic LTP [42]. Recently, a KA dependent presynaptic form of LTP has also been reported. Low frequency stimulation, paired with voltage clamps at postsynaptic sites induce NMDA receptor independent pre-LTP. KA GluK1 receptor is involved [42].

## KA receptor in LTD in the cortex and amygdala

Long-term depression (LTD) is another major form of plasticity that contributes to synaptic functions. In the ACC and IC, the induction of LTD requires activation of NMDA receptor or L-type voltage gated calcium channels or mGluRs ([37, 43, 44] for review). There is no report of the involvement of KA receptors in the induction of LTD. For the expression of LTD, it is likely mediated by reduced postsynaptic AMPA receptor functions, especially GluA2 subtype receptors in the ACC.

In the amygdala, low-frequency stimulation that usually induce LTD in the hippocampus did not induce LTD but LTP [41], and low-frequency stimulation paired with no postsynaptic depolarization can trigger the presynaptic form of LTP [42].

## LTD of KA receptor mediated responses

Although in the amygdala and ACC/IC, KA receptor related LTD has not been reported, there are reports of KA receptor LTD in the perirhinal cortex and hippocampus [45]. This form of KA receptor mediated LTD is induced by repetitive stimulation at 5 Hz, and NMDA receptor independent [46]. It has been suggested that this form of KA receptor mediated LTD may contribute to working memory.

## Roles of KA receptors in behavioral anxiety

Genetic studies in humans have suggested that certain types of KA subtype receptors may be linked to behavioral anxiety or depression [47]. Due to the limitation of selective compounds that inhibit or activate selective subtypes of KA receptors, basic mechanisms of how KA receptors contribute to these mood disorders remain unclear. The generation of gene knockout mice help us at a certain level for the potential functions of KA receptors. For example, in GluK2 KO mice, behavior fear is reduced along with the reduction of LTP in the amygdala induced by theta-burst stimulation (TBS) [33]. In GluK4 KO mice, reduce anxiety has also been reported [48]; and in mice with overexpression of GluK4 in the forebrain, behavioral anxiety is enhanced [49]. However, due to complexed effects of KA receptors at excitatory and inhibitory synapses; as well as presynaptic vs postsynaptic locations, it is difficult to determine the functions of selective subtypes of KA receptors even within the same central region. For example, gene deletion of KA GluK1 receptor leads to changes in the anxiety-like behaviors [33]. However, this may be due to changes in excitatory vs inhibitory transmission in the amygdala [6, 31] as well as potential changes in amygdala related structures.

Selective development of GluK1 receptor agonists and antagonists provide better evidence of GluK1 in the brain. A recent study has shown both genetically and pharmacologically that GluK1 is critical for presynaptic LTP in the ACC neurons [38]. This form of pre-LTP is absent in GluK1 KO mice as well as after inhibition of GluK1 receptor by UBP302. Furthermore, in mice exposed to a standard elevated plus maze (EPM) or a raised open platform, the pre-LTP was partially reduced or completely blocked. These results provide strong evidence that GluK1 dependent pre-LTP in the ACC may be involved in behavioral anxiety. Erasing pre-LTP by a pharmacological inhibitor of HCN channels produced inhibitory effects on injury induced anxiety [38].

### Possible functional link between KA receptor dependent LTP and LTD with behavioral anxiety

Anxiety is often long-lasting, if the environment or factors that contribute to the anxiety persist. It is thus expected that long-term changes in synaptic transmission of anxiety-related neurons or circuits may at least partially contribute behavioral anxiety-like responses. There are at least two major mechanisms for KA dependent LTP which may contribute to anxiety. One is to directly enhance neuronal responses to the same input signal such as visual, auditory or somatosensory. Consequently, neurons will fire more action potentials. Alternatively, synaptic LTP affects the jitter of neuronal action potentials (for example, see [50, 51]). As a result, neuronal activity will be altered. Such changes are not simply the increase or decrease of the amount of spikes. It is also possible that both mechanisms may take place in a certain subset of neurons, or neuronal circuits. KA-dependent plastic changes may provide a key synaptic basis for these neuronal circuit functions in the condition of anxiety. For LTD, similar KA-dependent mechanisms may apply, including the depression of excitatory and inhibitory transmission. Future studies using integrative approaches to explore molecular basis of neuronal activity with the anxiety-related neuronal circuits are clearly needed.

## Conclusion and future directions

Understanding the role of various molecular targets in anxiety disorders will help to address the etiology of anxiety and lead to development of novel treatments. Although many potential protein targets for the development of new anxiolytic compounds has been proposed, a few drugs are clinically effective for the treatment of anxiety in patients. KA receptor dependent pre-LTP presents a new mechanism for behavioral anxiety in the cortex, in addition to its modulatory effects on central synaptic transmission. Subtype selective chemicals targeted on KA receptors may provide potential new drugs for helping patients with anxiety, pain and depression.
